# What rationale do GPs use to choose a particular antibiotic for a specific clinical situation?

**DOI:** 10.1186/s12875-019-1068-7

**Published:** 2019-12-20

**Authors:** Jegatha Krishnakumar, Rosy Tsopra

**Affiliations:** 1 Université Paris 13, 74 rue Marcel Cachin, Bobigny, France; 2 Leeds Centre for Respiratory Medicine, St James’s University Hospital, Leeds, UK

**Keywords:** Antibacterial agents, General practice, Primary care, Clinical decision-making

## Abstract

**Background:**

Many studies have investigated the ways in which physicians decide whether to prescribe antibiotics, but very few studies have focused on the reasons for which general practitioners (GPs) choose to prescribe a particular antibiotic in a specific clinical situation. Improvements in our understanding of the rationale behind GPs’ decisions would provide insight into the reasons for which GPs do not always prescribe the antibiotic recommended in clinical practice guidelines and facilitate the development of appropriate interventions to improve antibiotic prescription.

The objective of the study was to understand the rationale used by GPs to decide which antibiotic to prescribe in a specific clinical situation, and to propose a model representing this rationale.

**Methods:**

We used a three-step process. First, data were collected from interviews with 20 GPs, and analysed according to the grounded theory approach. Second, data were collected from publications exploring the factors used by GPs to choose an antibiotic. Third, data were used to develop a comprehensive model of the rationale used by GPs to decide which antibiotic to prescribe.

**Results:**

The GPs considered various factors when choosing antibiotics: factors relating to microbiology (bacterial resistance), pharmacology (adverse effects, efficacy, practicality of the administration protocol, antibiotic class, drug cost), clinical conditions (patient profile and comorbid conditions, symptoms, progression of infection, history of antibiotic treatment, preference), and personal factors (GP’s experience, knowledge, emotion, preference).

**Conclusions:**

Various interventions, targeting all the factors underlying antibiotic choice, are required to improve antibiotic prescription. GP-related factors could be improved through interventions aiming to improve the GPs’ knowledge of antibiotics (e.g. continuing medical education). Factors relating to microbiology, pharmacology and clinical conditions could be targeted through the use of clinical decision support systems in everyday clinical practice.

## Background

Antibiotic misuse is a major public health problem worldwide. One sort of misuse is the prescription of the wrong antibiotic molecule by the physician [1]. Such errors expose patients to the unnecessary risk of adverse effects, complications, and death [2], and the population to the risk of resistant bacteria emerging [3].

Most antibiotics are prescribed by general practitioners (GPs) in primary care settings. GPs often experience difficulties deciding which antibiotic molecule to prescribe because:

- Antibiotic selection is a complex task in which many factors, such as clinical condition and tolerance in the patient, the microbiological and clinical efficacy of the antibiotic, ecological risk, and drug cost, must be taken into account [4].
- Antibiotic selection is mostly empirical, i.e. the antibiotic is selected without knowledge of the antimicrobial drug susceptibility profile of the causal pathogen [5, 6].
- Antibiotic selection is guided by bacterial resistance rates, which change frequently and rapidly, often without the awareness of physicians [7].

National health authorities release clinical practice guidelines [5, 8] to guide GPs in their choice of appropriate antibiotics. Clinical practice guidelines are textual documents written by groups of experts, who provide recommendations based on evidence from scientific publications. These recommendations are provided in the form of “clinical situation/antibiotic” associations, in which a particular antibiotic is recommended for a particular patient profile. For example, fosfomycin trometamol is the antibiotic recommended for first-line treatment of acute uncomplicated cystitis in women [5].

However, despite the extensive diffusion of clinical practice guidelines, GPs still frequently prescribe the wrong antibiotic molecule [9–11]. Indeed, they often choose to prescribe non-recommended, expensive broad-spectrum antibiotics (e.g. fluoroquinolones) rather than the first-line antibiotics recommended in clinical practice guidelines [12, 13]. There is a need to determine how GPs decide which antibiotic to prescribe, so that we can understand why they do not always prescribe the recommended antibiotics.

Many studies have investigated physicians’ decision-making processes for antibiotic prescription. Various factors have been identified [14–16]: (i) factors relating to the physician’s characteristics (e.g. clinical experience) and attitudes (e.g. fear, feelings of uncertainty, desire to satisfy patients), (ii) factors relating to the patient’s condition (e.g. allergy), symptoms and anxiety, and (iii) factors relating to the healthcare system (e.g. patients’ health insurance, public health policies). However, most of these studies have focused principally on the decision as to whether or not to prescribe antibiotics. Very few studies have focused on the reasons why GPs choose a particular antibiotic in a specific clinical situation. A better understanding of the rationale used by GPs in such situations would shed light on the reasons for which GPs do not always choose to prescribe the recommended first-line antibiotic, making it possible to develop appropriate interventions to improve antibiotic prescription.

In this study, the rationale used by GPs in their choice of a particular antibiotic for a specific clinical situation was investigated, and a model representing this rationale is proposed.

## Methods

We used a three-step process to explore the rationale used by GPs to choose a particular antibiotic for a specific clinical situation:

- Semi-structured interviews of GPs were conducted. This provided an initial list of the reasons why GPs choose a particular antibiotic in a clinical situation.
- A literature review of publications exploring the rationale used by GPs to choose an antibiotic was then conducted. This added to the first list of reasons derived from interviews with GPs, leading to a final list of possible reasons.
- Finally, this list was used to build a model representing the rationale used by GPs to choose an antibiotic.

### Step 1: data collected from interviews with general practitioners

Qualitative research methods were used, due to their suitability for exploring and understanding complex processes in detail [17]. Data were collected and analysed according to the grounded theory approach [17]. In this iterative process, data are collected and analysed simultaneously. At each iteration, the data are broken up, and marked with a meaningful code label. This process results in a list of codes, which are then classified into themes. These themes are then used to develop a theory explaining the problem studied. Throughout this process, a “constant comparison” approach was used, in which the codes and themes obtained for new data were continually compared with those obtained for the previously collected data.

#### Data collection

Semi-structured interviews were conducted by a junior doctor in training (JK).

The participants were GPs practicing in primary care. They were recruited by word of mouth, telephone calls, e-mails, and through the faculty of medicine. JK first contacted a few GPs met during medical training, and asked them both (i) if they would agree to be involved in the study, and (ii) if they could suggest other colleagues that we could contact. She then contacted these colleagues, and repeated the process until sufficient numbers of GPs had been recruited. We were careful to ensure that the panel of GPs had the greatest possible diversity of experience and opinions, by selecting GPs of both sexes, with a wide range of ages, and of durations and types of practice (public or private sector).

The interview guide was developed for this study, and tested with five junior doctors before its use with the GPs. During the interview, seven simulated clinical cases were presented orally to the GPs (see Additional file 1). These clinical cases concerned typical clinical situations frequently encountered in primary care: cystitis, pyelonephritis, prostatitis, pharyngitis, sinusitis, otitis and pneumonia. These cases should lead to the prescription of antibiotics, in accordance with French clinical practice guidelines [18–20]. For each clinical case, a series of questions were asked, relating to the choice of antibiotic, the strategy underlying this choice and the factors influencing antibiotic choice. GPs were also asked whether there were other situations in which they would prescribe a different antibiotic molecule and the reasons for which they would do so. The questions posed were open-ended, to encourage GPs to discuss the issues freely.

An audio recording was made, transcribed and rendered anonymous for each interview. The GP interviewed decided on the location of the interview: the GP’s home or office, the faculty of medicine or by telephone.

Interviews were conducted until a theoretical saturation point, corresponding to the point at which no new ideas emerged from interviews, was reached.

#### Data analysis

The data obtained from interviews were analysed manually, and independently, by JK and RT. For each interview, the verbatim was first broken down into words or sentences to obtain a list of codes representing the information contained in the data. These codes were then grouped into subcategories, categories and themes. This resulted in a first list of codes.

### Step 2: data collected from scientific publications

#### Data collection

We added to the initial list of codes derived from the interviews of GPs, by searching for publications exploring the factors used by GPs to choose an antibiotic. The following MeSH query was used in the MEDLINE database:

(“Family Practice” OR “Physicians, Primary Care” OR “Physicians, Family” OR “Ambulatory Care” OR “General Practice”)

AND

(“Practice Patterns, Physicians” OR “Clinical Decision-Making” OR “Decision Making/drug therapy” OR “Decision Making/therapy”)

AND

(“Anti-Bacterial Agents/therapeutic use” OR “Anti-Infective Agents/therapeutic use”)

We retrieved and reviewed 230 abstracts. Studies were considered eligible for inclusion if they satisfied the following criteria:

- Focusing exclusively on GPs practicing in primary care
- Exploring factors relating to the choice of antibiotic molecule
- Written in English or French

#### Data analysis

The scientific publications selected from the literature review were analysed manually, and independently by JK and RT. For each publication, the factors involved in antibiotic choice were extracted. These factors were then incorporated into the list of codes derived from the interviews of GPs. This resulted in a final list of codes.

### Step 3: model representing the rationale used by GPs to choose an antibiotic in a clinical situation

The list resulting from step 2 was used to develop a comprehensive model of the rationale used by GPs to decide which antibiotic to prescribe. The hierarchical relationships between codes, identified in steps 1 and 2, but also the relationships of each code to one of the four main entities involved in antibiotic decision (i.e. the patient, the doctor, the bacterium, or the antibiotic), were used to build the model.

At each step of the method described, JK and RT compared their analyses and discussed inconsistencies until a consensus was obtained.

The results of steps 1 and 2 are presented together in the results, whereas the model resulting from step 3 is presented separately.

## Results

### Characteristics of GPs and scientific publications

Interviews with 20 GPs and the data from three publications [21–23] were analysed.

Three GPs were contacted, but refused to be involved in the study because they lacked time. The 20 GPs interviewed had a mean age of 47 years (range: 27 to 70 years). They had been practicing in the primary care setting for a mean of 18 years (range: 2 months to 37 years). Thirteen were salaried (public sector), and seven worked in private practice. Twelve were women. All but one of the GPs were interviewed with the same seven clinical cases (see Additional file). The remaining GP was interviewed with only five of the seven clinical cases because of a lack of time. Interviews lasted between 15 and 35 min.

One of the publications studied [21] investigated the reasons underlying antibiotic choice by GPs, but focused on broad-spectrum and fluoroquinolone antibiotics. The other two publications [22, 23] did not address the problem of antibiotic choice as a primary objective, but they reported findings regarding the factors involved in antibiotic choice.

### Antibiotic choice is guided by four main factors

Our analysis of GP interviews and scientific publications (steps 1 and 2) revealed that antibiotic choice was guided by four main factors: the probable causal bacteria, the patient’s condition, antibiotic properties and general practitioner-related factors.

#### Antibiotic choice is guided by the probable causal bacteria

Almost all the GPs reported that they chose the antibiotic to prescribe according to the bacterium causing the infection *(Ex1)*. Identification of the causal bacterium is not easy in primary care, because GPs cannot necessarily perform bacterial tests during consultations. They therefore have to use the patient’s symptoms and epidemiological data (e.g. the prevalence of causal agents) to formulate hypotheses concerning the most likely causal bacterium *(Ex2)*. The GPs then choose an antibiotic to which they presume the bacterium is susceptible.

*Ex1 (from publication* [21]*): “The likely infecting organism was also reported as a major influence on which antibiotic to prescribe”.*

*Ex2: “For otitis… if there is also conjunctivitis, then I prescribe amoxicillin clavulanic-acid, because I suspect the pathogen to be Haemophilus influenzae”.*

In some cases, GPs may decide to confirm their hypotheses, by prescribing bacteriological tests (e.g. urine culture). However, as the results of these tests may take some time to obtain, GPs are nevertheless obliged to prescribe antibiotics in accordance with their hypothesis, before subsequent readjustment, if necessary, on the basis of bacteriological tests *(Ex3)*.

*Ex3: “For uncomplicated pyelonephritis… I think I would prescribe a urine culture test, and then adjust the prescription according to the result of the test (…). I first prescribe ofloxacin, because of its broad-spectrum and then readjust after 48 hours, according to the results of the bacterial test. For example, if the bacterium is susceptible to amoxicillin, I would readjust and prescribe amoxicillin. But, without the result of the bacterial test, I would never prescribe amoxicillin because of the risk of resistance, unlike fluoroquinolones for which susceptibility is higher.”*

A few GPs also reported a preference for prescribing antibiotics likely to do little collateral damage (i.e. those not known to generate bacterial resistance) *(Ex4)*.

*Ex4: “I now prescribe fluoroquinolones as a first-line treatment, to avoid the emergence of extended-spectrum beta-lactamases”.*

#### Antibiotic choice is guided by the patient’s condition

All GPs reported taking patient profile (age, allergy, pregnancy), medical history and comorbid conditions (e.g. renal failure) into account. For example, for frail elderly patients or patients with comorbid conditions likely to worsen the infection (e.g. diabetes), the GPs preferred to prescribe antibiotics that were “powerful” or taken in long courses *(Ex5, Ex6)*.

*Ex5: “For pneumonia in young people I give amoxicillin (...). For elderly people, I prefer to give amoxicillin/clavulanic acid, which is more efficient, especially after flu-like conditions, which often leave patients frailer”.*

*Ex6: “For acute cystitis, I usually give Monuril® (…). But today, I saw a patient who had an history of pyelonephritis. Because of this antecedent, I was worried about prescribing Monuril® as a single-dose… So, I decided to prescribe lomefloxacin for three days. So yes, because of her medical history, I wanted to be more effective than the usual treatment”.*

All GPs also said that they took the patient’s symptoms and the course of the infection into account when prescribing antibiotics. For example, they reported a preference for broad-spectrum antibiotics (e.g. fluoroquinolones) for serious, intense, risky, persistent, repeated or complicated infections *(Ex7, Ex8)*.

*Ex7: “For otitis… if symptoms are severe I prescribe Oflocet®, otherwise I give Augmentin®”.*

*Ex8: “For childhood pharyngitis… Amoxicillin as the first-line treatment (…). But if the patient’s condition deteriorates, then I add clavulanic acid, because I want to be more effective and to cover more of the pathogens likely to cause upper respiratory tract infections”.*

Some GPs also said that they took the patient’s history of antibiotic treatment into account. They avoid prescribing antibiotics that had not proved effective in the patient in the past, and antibiotics already prescribed to the patients in the last few months, so as to prevent the occurrence of bacterial resistance *(Ex9)*.

*Ex9: “For prostatitis … I avoid fluoroquinolones (…) if the patient has taken fluoroquinolones in the last three months”.*

Patient preferences were also taken into account. GPs said that they sometimes adjusted their prescriptions according to the patient’s preference in terms of the type of molecule, galenic formulation or mode of administration *(Ex10, Ex11)*.

*Ex10: (from publication* [21]*): “Many GPs explained how fluoroquinolones were popular with a range of patients due to the low incidence of side-effects and the twice daily dose”.*

*Ex11: “I listen a little to what the patient says… if the patient repeatedly had sinusitis and tells me that one particular antibiotic is efficient, then I prescribe this antibiotic if it is appropriate. So, yes, sometimes, the patient’s wishes may play a role in antibiotic choice”.*

#### Antibiotic choice is guided by antibiotic properties

Most GPs reported that they considered the efficacy of the antibiotic when making their choice. They reported a preference for antibiotics with marketing authorisation for the infection, good pharmacokinetic parameters (e.g. rapid action), and antibiotics known to treat the type of infection concerned effectively *(Ex12, Ex13)*.

*Ex12: “For sinusitis… I prescribe Orelox®, because it has always been effective”.*

*Ex13: “For prostatitis …. I give ofloxacin… This antibiotic reaches high concentrations in the urinary tract, and this is important because prostatitis is a deep infection”.*

Many GPs also said that they took the adverse effects of antibiotics into account *(Ex14)*. They avoided prescribing antibiotics known to have adverse effects (e.g. fluoroquinolone-induced Achilles tendinitis, the adverse gastrointestinal effects of amoxicillin clavulanic-acid).

*Ex14: “For pharyngitis… I prescribe amoxicillin (...). It is very effective, and it has the least side effects”.*

The administration protocol was often taken into account. Antibiotics with a convenient administration protocol (i.e. low daily dose, short-course treatment) were preferred, to maximise patient observance *(Ex15)*. Administration route, galenic formulation and the flavour of the preparation may also be adjusted to patient profile, to encourage patients to take their treatment *(Ex16)*. For example, if the patient has trouble swallowing tablets, antibiotics may be prescribed as syrups or suspensions, or for intramuscular injection.

*Ex15: “For cystitis… I prescribe Monuril® as a single dose (…). It is very convenient for patients”.*

*Ex16: “Josacin® is highly suitable for use in children because of its strawberry taste”.*

Some GPs also explained that they avoided prescribing antibiotics from classes considered to be precious, such as fluoroquinolones, and third-generation cephalosporins. They reserved these antibiotics for very serious cases and for cases of infection with highly resistant bacteria *(Ex17, Ex18)*.

*Ex17 (from publication* [21]*) “They needed to keep broad-spectrum antibiotics in reserve for severely ill patients”.*

*Ex18: “For pharyngitis, I avoid third-generation cephalosporins. We have to preserve them.”*

Very few GPs reported taking cost into account in their choice of antibiotic. Those that did considered the cost of the drug itself *(Ex19)*, but also the costs relating to hospital admission that antibiotic administration might prevent.

*Ex19: “I won’t prescribe pristinamycin as a first-line treatment because it is expensive for our national health insurance”.*

#### Antibiotic choice is guided by general practitioner-related factors

Antibiotic choice is also guided by the GPs’ antibiotic knowledge *(Ex20)*, habits, experience *(Ex21)*, and preferences *(Ex22)*. These preferences seem to differ between GPs.

*Ex20: “For pyelonephritis… I used to prescribe quinolones because that was what we prescribed during my medical training, but I think things are changing…”.*

*Ex21: “For cystitis… Monuril® (…). This is the antibiotic recommended for first-line treatment, and I have also had good experiences with this drug in terms of side effects and efficacy.”*

*Ex22: “I know we can prescribe other antibiotics in this situation, but I prefer Orelox®.”*

All GPs said that they used external resources to guide their choice of antibiotic. Clinical practice guidelines were the most frequently mentioned resource, followed by antibiotic websites, personal memos, and scientific publications *(Ex23)*. GPs were also found to be influenced by the practices of colleagues, and of specialists in hospitals *(Ex24)*. One GP reported being influenced by sales representatives from the pharmaceutical industry.

*Ex23: “I follow the recommendations in clinical practice guidelines (...) I also update my knowledge with the antibiotic website”.*

*Ex24 (from publication* [21]*): “GPs’ prescribing choices were also influenced by discussions with other GPs at practice meetings”.*

Half the GPs also reported that they followed their own instincts when choosing antibiotics *(Ex25),* and one even admitted that antibiotic choice was influenced by his/her mood at the time of prescription *(Ex26)*.

*Ex25: “For cystitis… instinctively, I prescribe Monuril®”.*

*Ex26: “For sinusitis… I give Augmentin® or Zinnat®, depending on my mood”.*

Some GPs also said that they were guided by their fears *(Ex27)* and by feelings of responsibility for their patients *(Ex28)*. They explained that they sometimes prescribed very powerful antibiotics not recommended in guidelines because they were concerned about the possibility of complications occurring in the patient and because they wanted to do their best for their patients.

*Ex27: “For pneumonia… I should give amoxicillin, but this is the only situation in which I prescribe amoxicillin-clavulanic acid. I prefer to be more effective. Pneumonia is a source of anxiety for doctors, and for this reason, doctors may deliberately decide to prescribe outside of the guidelines”.*

*Ex28 (from publication* [21]*): “Most of them justified their current liberal prescribing of fluoroquinolones on the basis of their duty to do the best for “the patient in front of them””.*

### Model of the rationale used by general practitioners in their choice of antibiotic

**Fig. 1:**
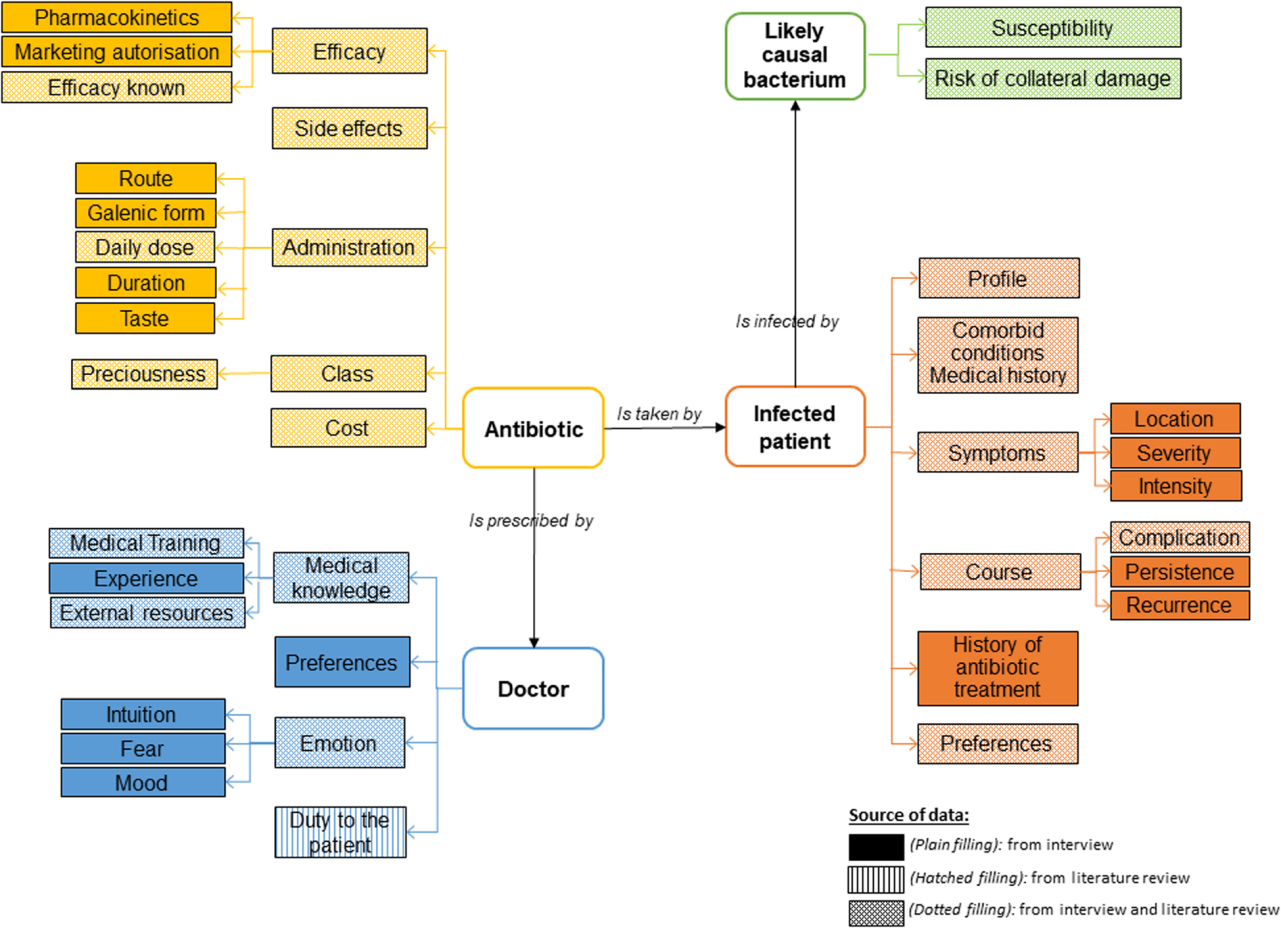
Model of the rationale used by GPs for antibiotic choice. Various factors were considered: factors relating to microbiology, pharmacology, clinical conditions and personal factors.

The model resulting from our analysis, including all the factors involved in antibiotic choice, is presented in Fig. 1.

## Discussion

We conducted a qualitative research study, to explore the rationale used by GPs in their choice of an antibiotic in a specific clinical situation. We showed that GPs considered various factors when selecting the antibiotic to prescribe: factors relating to microbiological aspects (e.g. bacterial resistance), pharmacology (e.g. antibiotic properties), clinical conditions (e.g. symptoms), and personal factors (e.g. experience, knowledge).

### Strengths and limitations

A model representing the rationale underlying the choice of antibiotics by GPs is proposed here. The exactness and exhaustiveness of the model were optimised by: (i) using open-ended questions, to encourage GPs to discuss their reasons for choosing a particular antibiotic more freely, (ii) conducting interviews until data saturation was achieved (obtained after the 17th interview), (iii) having two individuals with a medical background analyse the data separately, and then together, until a consensus was reached, (iv) complementing the model with the results of relevant published studies.

This work had several limitations. First, the interviewer was a junior doctor in training, not used to conducting interviews. We tried to overcome this limitation by training the junior doctor before the interviews were conducted (e.g. through the testing of the interview guide with five junior doctors). Each interview was also discussed, providing the junior doctor with feedback. The number of GPs interviewed may also seem not enough, but data saturation was achieved after the 17th interview. This may be explained by the large number of clinical cases that GPs were asked to consider. A “social desirability bias” [24] may also have been introduced, because GPs may have reported practices not corresponding to their real actions, due to fears of being judged or a desire to appear more responsible than they really were. This bias may have been increased by the fact that the interviewer was a junior doctor. However, we tried to limit this bias by reassuring the GPs that their anonymity would be preserved, and by asking them to give answers corresponding to their usual clinical practices.

### Comparison with published findings

We studied the factors considered by GPs during antibiotic choice. Other studies [25–29] have also investigated these factors, but for other medical specialities. Some factors, such as the risk of collateral damage, patient preferences, course of infection, antibiotic class (precious or not), specialist advice, and the mood, instinct and experience of GPs, were retrieved only in our study. This may be because some of these factors are specific to GPs (e.g. patient preferences), or likely to be highlighted only in qualitative studies (e.g. instinct, mood of the physician). By contrast, some factors retrieved in other studies were not picked up here: “*medical speciality*”, “*patient origin*” (e.g. ethnic group [27], home area [11]), “*drug interactions*” [25], “*environmental and economic factors*” (e.g. unhygienic environment [29], availability of antibiotics [28], pressure of national health authorities [26] and health insurance [11]). “Medical speciality” and “patient origin” could not be retrieved, by definition, because of the study design (all the interviewees were GPs practicing in the Parisian region). Similarly, “environmental and economic factors” appear to be specific to certain countries. Surprisingly, the factor “drug interaction” was not retrieved in our study.

### Implications for the establishment of interventions to improve antibiotic prescription

We found that GPs did not limit antibiotic prescription to the molecules recommended in clinical practice guidelines. They used a rationale based on factors relating to the causal bacteria, the patient, the antibiotic and personal factors. This rationale can be broken down into two components [30]:

(i) An intuitive element corresponding to GP-related factors, such as knowledge, experience, and memory. This intuitive element makes it possible for the GP to come up with a quick “automatic” response on the basis of experience with similar situations [30].
(ii) An analytical element based on factors relating to the patient, antibiotic and bacteria. This analytical element provides a slower, more rational answer based on the checking of a series of hypotheses [30].

GPs initially use the intuitive element to find an immediate answer (e.g. *if uncomplicated cystitis in women, then fosfomycin*), which is then checked by the analytical system (e.g. *is fosfomycin contraindicated in this patient?*). An incorrect balance between these two elements may lead to incorrect decisions, such as the prescription of an antibiotic that is not recommended.

These findings may facilitate the design of interventions to improve antibiotic prescription. Interventions should focus on reducing the number of antibiotics prescribed wrongly (e.g. prescription of antibiotics for a viral infection), but also on improving the quality of prescriptions in terms of antibiotic choice (e.g. avoiding broad-spectrum antibiotics). Our findings suggest that interventions for improving antibiotic choice should target both the intuitive and analytical systems. The intuitive elements, corresponding to the knowledge and experience of GPs, could be targeted through interventions to improve GPs’ knowledge about antibiotics [31, 32], through continuing medical education, practice meetings with colleagues, or the production of clinical practice guidelines easier to use in everyday clinical practice. The analytical elements, corresponding to the checking of properties relating to the causal bacteria, the antibiotic and the patient’s condition, could be improved through the use of clinical decision support systems. A clinical decision support system displaying the properties of antibiotics to GPs could help them to make more appropriate choices during prescription [33–38]. For example, the visualisation of antibiotic activity spectra could provide GPs with information about the current level of bacterial resistance, making it easier to choose the most appropriate antibiotic. Likewise, the visualisation of modes of administration could help GPs to choose the most convenient antibiotic for their patients [39]. Clinical decision support systems should display all the properties described here, to provide GPs with all the information they need during prescription, which would favour their adoption by GPs. These properties should be displayed in a usable interface, allowing GPs to view the properties of antibiotics at a glance [35, 38], and they should be updated regularly through external resources [40–42] (e.g. microbiological observatories).

## Conclusions

When GPs decide which antibiotic to prescribe, they do not limit themselves to those recommended in clinical practice guidelines. They use their knowledge and experience to guide their choice of antibiotic, but they also take into account factors relating to microbiology, pharmacology, and clinical conditions. All these factors should be considered when establishing interventions to improve antibiotic prescription.

## Supplementary information

**Additional file 1.** Tsopra_ Interview guide, Clinical cases and the questions the GPs were asked during interviews.

## Data Availability

The datasets generated and/or analysed during the current study are not publicly available due to data confidentiality related to the funding source but are available from the corresponding author on reasonable request.

## Abbreviation

GPs: General practitioners
