# Supplementary material for: What rationale do GPs use to choose a particular antibiotic for a specific clinical situation?
Source: BMC Fam Pract. 2019 Dec 20;20:178. doi: 10.1186/s12875-019-1068-7 (PMC6925435; doi:10.1186/s12875-019-1068-7)
Supplement: Supplementary file 1 — Additional file 1. Tsopra_ Interview guide, Clinical cases and the questions the GPs were asked during interviews. [file 12875_2019_1068_MOESM1_ESM.docx]

# **Additional file**

**File name:** Tsopra_AdditionalFile1

**Title of data:** Interview guide

**Description of data:** Clinical cases and the questions GPs were asked during interviews.

Seven clinical cases were presented orally to the GPs (see below). The GPs had to decide which antibiotic should be prescribed, in accordance with French clinical practice guidelines.

**Pharyngitis:**

You see in consultation a four-year-old child who has had a fever and sore throat since the previous day. During your examination, you note inflammation of the tonsils and pharynx. You perform the rapid antigen detection test, which gives a positive result. You diagnose group A streptococcal pharyngitis.

**Sinusitis:**

You see in consultation a 24-year-old man who has had fever and intense right maxillary pain for two days. He has taken a symptomatic treatment for a cold for three days. He also has purulent rhinorrhoea. You diagnose uncomplicated right maxillary sinusitis.

**Otitis:**

You see in consultation a three-year-old child who has had a fever (temperature: 40°C) with a strong right otalgia for two days. You diagnose acute purulent otitis of the right ear.

**Pneumonia:**

You see in consultation a 40-year-old man with a fever, cough with purulent sputum and chest pain. On clinical examination, you hear crackles at the base of the left lung. You diagnose acute community-acquired pneumonia.

**Prostatitis:**

You see in consultation a 60-year-old man with frequent urination, a burning sensation on urination, and fever of two days’ duration. The urine dipstick test gives a positive result for leukocytes and nitrites. You diagnose simple acute prostatitis.

**Cystitis:**

You see in consultation a 20-year-old woman complaining of a burning sensation on urination for the last two days, without fever or lumbar pain. The urine dipstick test gives a positive result for leukocytes and nitrites. You diagnose acute uncomplicated cystitis.

**Pyelonephritis:**

You see in consultation a 35-year-old woman, complaining of a burning sensation on urination with fever and left lumbar pain. The urine dipstick test gives a positive result for leukocytes and nitrites. You diagnose acute uncomplicated pyelonephritis.

For each clinical case, the questions asked were:

Which treatment would you prescribe?

Why would you choose this antibiotic?

Are there any other reasons for choosing this antibiotic?

Are there any other related situations for which you would prescribe another molecule?

If yes: why would you choose this molecule?
